# Profiling endogenous adrenal function during veno-venous ECMO support in COVID-19 ARDS: a descriptive analysis

**DOI:** 10.3389/fendo.2023.1321511

**Published:** 2024-01-25

**Authors:** Clemens Baumgartner, Peter Wolf, Alexander Hermann, Sebastian König, Mathias Maleczek, Daniel Laxar, Marko Poglitsch, Oliver Domenig, Katharina Krenn, Judith Schiefer, Alexandra Kautzky-Willer, Michael Krebs, Martina Hermann

**Affiliations:** ^1^ Department of Internal Medicine III, Medical University of Vienna, Vienna, Austria; ^2^ Department of Internal Medicine I, Medical University of Vienna, Vienna, Austria; ^3^ Department of Anaesthesia, Intensive Care Medicine and Pain Medicine, Medical University of Vienna, Vienna, Austria; ^4^ Ludwig Boltzmann Institute for Digital Health and Patient Safety, Vienna, Austria; ^5^ Attoquant Diagnostics, Vienna, Austria

**Keywords:** cortisol, critical illness related corticosteroid insufficiency, extracorporeal membrane oxygenation, acute respiratory distress syndrome, severe coronavirus disease 2019

## Abstract

**Background:**

Prolonged critical illness is often accompanied by an impairment of adrenal function, which has been frequently related to conditions complicating patient management. The presumed connection between hypoxia and the pathogenesis of this critical- illness- related corticosteroid insufficiency (CIRCI) might play an important role in patients with severe acute respiratory distress syndrome (ARDS). Since extracorporeal membrane oxygenation (ECMO) is frequently used in ARDS, but data on CIRCI during this condition are scarce, this study reports the behaviour of adrenal function parameters during oxygenation support with veno-venous (vv)ECMO in coronavirus disease 2019 (COVID-19) ARDS.

**Methods:**

A total of 11 patients undergoing vvECMO due to COVID-19 ARDS at the Medical University of Vienna, who received no concurrent corticosteroid therapy, were retrospectively included in this study. We analysed the concentrations of cortisol, aldosterone, and angiotensin (Ang) metabolites (Ang I–IV, Ang 1–7, and Ang 1–5) in serum via liquid chromatography/tandem mass spectrometry before, after 1 day, 1 week, and 2 weeks during vvECMO support and conducted correlation analyses between cortisol and parameters of disease severity.

**Results:**

Cortisol concentrations appeared to be lowest after initiation of ECMO and progressively increased throughout the study period. Higher concentrations were related to disease severity and correlated markedly with interleukin-6, procalcitonin, pH, base excess, and albumin during the first day of ECMO. Fair correlations during the first day could be observed with calcium, duration of critical illness, and ECMO gas flow. Angiotensin metabolite concentrations were available in a subset of patients and indicated a more homogenous aldosterone response to plasma renin activity after 1 week of ECMO support.

**Conclusion:**

Oxygenation support through vvECMO may lead to a partial recovery of adrenal function over time. In homogenous patient collectives, this novel approach might help to further determine the importance of adrenal stress response in ECMO and the influence of oxygenation support on CIRCI.

## 1 Background

Severe acute respiratory distress syndrome (ARDS) often demands support via extracorporeal membrane oxygenation (ECMO) to maintain vital organ function (1). Due to disease severity, affected patients are frequently prone to prolonged critical illness, which subsequently leads to dysfunction of the hypothalamic–pituitary–adrenal (HPA) axis (2). As concomitant failure of maintaining an autonomous stress response, critical- illness- related corticosteroid insufficiency (CIRCI) is often associated with the continued requirement of vasopressor support, electrolyte disturbances, and poor neurological recovery post sedation (3). In case of primary adrenal insufficiency, co-evaluation of the renin–angiotensin–aldosterone system (RAAS) activity in these patients was previously proposed to improve reliable interpretation (4).

Since HPA-axis-perturbing hypoxia and inflammation are considered causes of CIRCI (5), the investigation of adrenal function in the setting of ECMO due to respiratory failure seems imperative. Recent studies reported the occurrence of CIRCI in patients during ECMO support (6, 7). However, due to different underlying causes of ARDS and the requirement of HPA axis-affecting medication like glucocorticoids, an accurate and consecutive assessment of adrenal function over time remains challenging. Severe coronavirus infectious disease 2019 (COVID-19) features a distinct type of ARDS (8). Commonly observed during the pandemic between 2020 and 2022, COVID-19 ARDS frequently served as a disease model for pulmonary failure, in which veno-venous (vv)ECMO represents the main therapy method to support lung function (1).

This study aimed to profile autonomous adrenal function under the requirement for vvECMO in prolonged COVID-19 ARDS and relate endogenous cortisol concentration to clinical features associated with CIRCI during the first 2 weeks of ECMO support. Focusing on long-term critical illness in this setting enables to investigate a homogeneous collective of intensive care patients with respiratory failure, which may also provide further insights into the pathophysiology of adrenal dysfunction in classical non-COVID ARDS.

## 2 Methods

To assess adrenal function during vvECMO support, we retrospectively evaluated a cohort of patients with respiratory failure treated at our centre between January 2020 and May 2021 (9). Previously collected data consisted of information about patients general demographics at admission, comorbidities, length of ICU stay at our centre, and disease course (including simplified acute physiology score (SAPS) 3 before vvECMO administration, vasopressor support, dialysis, and non-ECMO related complications), daily blood analyses for routine clinical care, which were conducted at the laboratory of our centre (10), and if the cause of respiratory failure was COVID-19. Along with other comorbidities associated with increased severity of COVID-19 (arterial hypertension, chronic heart disease, and chronic respiratory disease), the reported metabolic disorders were diagnosed type 2 diabetes and obesity (defined as BMI > 30 kg/m^2^). Daily observations regarding respiration included respiratory rate, Horovitz index, the type of ventilatory support, tidal volume, PEEP and peak inspiratory pressure, routine blood gas analyses, prone positioning, and the requirement for neuromuscular blockage, whereby the parameters of mechanical ventilation for each observation were presented as the mean value of the respective study day. The protective ventilatory strategy used within our patient’s collective was previously described (9). ECMO-specific data contained the duration of critical illness before ECMO (considered as the duration of invasive mechanical ventilation (IMV) before ECMO initiation), duration of ECMO in days, mean blood, and gas flow throughout the time of ECMO-support, and ECMO-related complications. For each patient, complications were recorded until the 28th day of ICU stay, the end of ECMO therapy, or death, whichever occurred first. Of the deceased patients, the time between ICU admission and death and cause of death were reported, which were recorded until the end of hospital stay.

### 2.1 Study population and specific blood sampling

Of the observed population, we included all patients receiving vvECMO, in which the underlying cause of ARDS was COVID-19. General exclusion criteria comprised continuous treatment with glucocorticoids (GC) or other medication potentially influencing HPA-axis signalling, i.e., etomidate, or azole antimycotics (11). Furthermore, we excluded all time points in which patients received single doses of GC treatment within 24 h before blood sampling to enable a reliable determination of adrenal function. Subsequently, we determined total serum cortisol (as the most relevant surrogate parameter for adrenal sufficiency) and serum aldosterone as our primary endpoints prior to (“pre ECMO”), after the first day (“acute “— i.e., between 16 and 24 h), 1 week after, and 2 weeks after vvECMO initiation. If vvECMO support ended before the observation period of 2 weeks, subsequent time points were excluded as well.

At each occasion along the specified timeline, the presence of CIRCI was determined according to the Society of Critical Care Medicine and European Society of Intensive Care Medicine, by a random cortisol measurement of <10 mcg/dL, i.e., 275.86 nmol/L (12). During ECMO, the present serum samples used to determine cortisol concentrations were collected daily approximately 7 a.m. and immediately stored at −80°C at our biobank. Regarding the assessment of concentrations, samples were spiked with deuterated internal standards for aldosterone (Aldosterone D4) and cortisol (Cortisol D3), and enriched target analytes were quantified by liquid chromatography/tandem mass spectrometry analysis following a solid-phase extraction (C18, Waters)-based sample preparation process. In cooperation with Attoquant diagnostics GmbH, data of RAS fingerprints (13–15) were used to depict RAAS activity along the sampling timeline, which included concentrations of angiotensin (Ang) I, II, III, IV, Ang 1–5, Ang 1–7, and the angiotensin-based marker for plasma renin activity (PRA-S), calculated as the sum of Ang I and Ang II (16, 17). Aldosterone values lower than 20 pmol/L, which could not be re-determined, were arbitrarily set to 10 pmol/L for a more reliable estimation of concentration.

### 2.2 Statistical analysis

Qualitative parameters are presented as count and percentage. Quantitative parameters are presented as median and interquartile range (IQR), and Kruskal–Wallis test was used for comparison. Data are represented in tables, and for the comparison of values between different observations along the specified timeline, the overall p-value is shown, whereby significance was determined by a two-sided α of < 0.05. Due to the exploratory design of our study, no correction for multiple testing was performed. Boxplots were used to depict cortisol and aldosterone concentrations at each occasion during the observed time course. Furthermore, Spearman correlation coefficients and scatter plots were used to determine noticeable correlations between cortisol and general ICU parameters and biomarkers regarding patient’s condition during vvECMO support. RAS fingerprints and relations between PRA-S and aldosterone were investigated in a subset of patients for which all required data on RAAS activity were present. For the illustration of RAS fingerprints, angiotensin metabolite levels below the lower limit of quantification were set to their lower limit of quantification. The relation between PRA-S and aldosterone was depicted by scatter plots and Spearman coefficients of log- transformed parameters using their natural logarithms. The R software (R Core Team. R: A Language and Environment for Statistical Computing. Vienna, Austria: R Foundation for Statistical Computing, 2020. https://www.R-project.org/.) was used for descriptive and analytical statistics.

## 3 Results

Our database contained data of 42 ICU patients in need of ECMO due to respiratory failure between January 2020 and May 2021 with samples stored at our biobank. Following our inclusion and exclusion criteria, unbiased cortisol and aldosterone values were available from 28 samples of 11 patients (10 male and 1 female) undergoing vvECMO due to COVID-19 ARDS. Of those, data on RAS fingerprints were available from a subset of 15 samples. Due to the low number of suitable parameters before ECMO, correlation analyses were only conducted for the observations after ECMO initiation. If not explicitly stated, described calculations were not significant.

### 3.1 Disease course during the observation period

Demographic data and present comorbidities at the time of study inclusion are enlisted in **Table 1**: overall, patients represented with a median age of 62 (53–66) years and were overweight, showing also a cumulation of type 2 diabetes, arterial hypertension, a high SAPS3 score, and severe ARDS according to Horovitz Index. None of the participants had a history of chronic kidney disease or received continuous immunosuppression. Median duration of critical illness before vvECMO initiation was 10 (8–12) days. All patients required mechanical ventilation until ECMO, whereby the majority received biphasic positive airway pressure (BIPAP) support. Tidal volume, PEEP, and peak inspiratory pressure before ECMO initiation were 354 (284–410) mL, 13 (12–14) cmH_2_O, and 29 (18–27) cmH_2_O, respectively. During ECMO, respiration was primarily managed via BIPAP, whereby in all patients, neuromuscular blockage was required during 7 (2–10) days. The median time of vvECMO support of our population was 21 (13–29) days. Blood and gas flow were 3.71 (3.52 –4.03) L/min and 4.04 (3.05 –4.72) L/min, respectively. After vvECMO initiation, a total of 10 patients needed vasopressor support within 15 (10 –21.5) days during the observation period. Two of them received haemodialysis for 3 and 19 days. The type and quantity of ECMO- and non-ECMO- related complications are reported in **Table 1**. Within the observation period, none of the patients developed bleeding related to ECMO cannulation, intracranial bleeding, or ischaemic stroke. Overall length of stay at our ICU was 26 (22–44) days. Five patients died, two after 18 and 45 days due to multi-organ failure, and three after 21, 23, and 31 days due to refractory COVID-19 ARDS despite vvECMO.

**Table 1:** Baseline characteristics of the observed study population and quantity of complications during the observation period.

	*N=11*
Age, years	62 (53–66)
Sex:	
female	1 (9%)
male	10 (90%)
BMI, kg/m^2^	27.8 (25.7– 29.8)
ICU-length of stay, days	26 (22–44)
Ventilation-free days within the first 28 days	0 (0–7)
SAPS3 score	68.0 (56.5– 74.5)
Horovitz index (p_a_O_2_/F_i_O_2_)	93.7 (77.2– 159.0)
Duration of critical illness before ECMO, days	10 (8– 12)
Death at ICU	5 (45%)
Mean ECMO-blood flow, L	3.71 (3.52– 4.03)
Mean ECMO-gas flow, L	4.04 (3.05– 4.72)
ECMO-duration, days	20.9 (13.0– 29.1)
Arterial hypertension, n	6 (55%)
Chronic heart disease, n	3 (27%)
Obesity, n	2 (18%)
Type-2 diabetes, n	6 (55%)
Chronic respiratory disease, n	2 (18%)
General bleeding	3 (27%)
Gastrointestinal bleeding	1 (9%)
Airway bleeding	5 (45%)
Pericardial effusion	3 (27%)
Haematothorax	1 (9%)
Pulmonary embolism	3 (27%)
Pneumothorax	1 (9%)

All qualitative parameters are given as count (percentage), and all quantitative parameters are given as median (IQR). ECMO blood and gas flow for each patient were recorded as the mean value observed over the time of ECMO support. Duration of critical illness before ECMO was considered as duration of invasive mechanical ventilation before ECMO.

### 3.2 Adrenal function during vvECMO support

**Figure 1:**
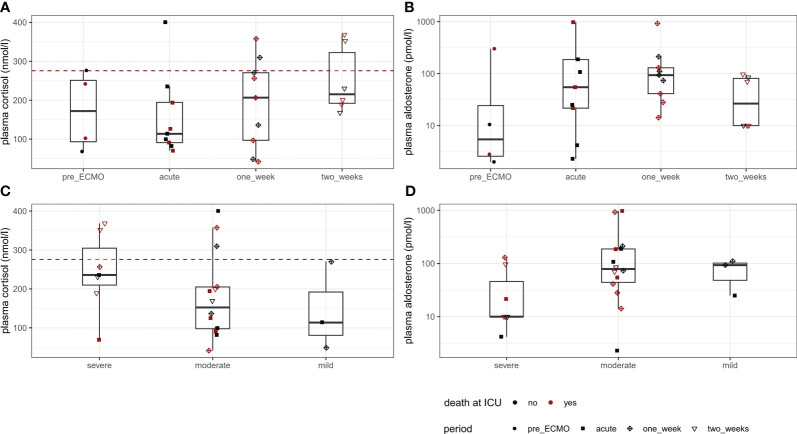
Serum cortisol and aldosterone concentrations throughout the observation period. Boxplots are depicted for concentrations at each observed point in time **(A, B)** and according to ARDS severity for all available measurements before and during vvECMO **(C, D)**. As shown by the red dashed line in **(A), (C)**, CIRCI was present in most individuals immediately before and during ECMO support.

**Figure 2:**
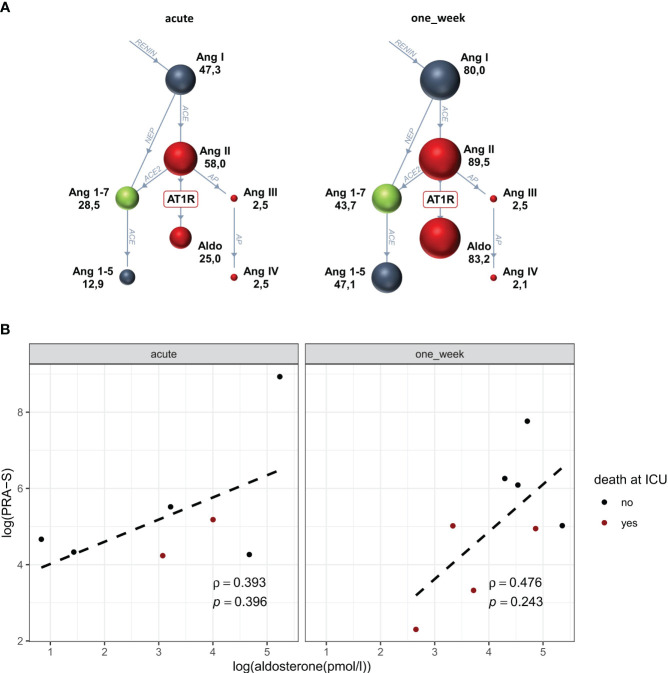
Renin–angiotensin–aldosterone system activity during the acute phase and after one week of vvECMO support. Within the subgroup analysis, angiotensin levels (shown as median) did not differ between the acute phase (n = 7) and after 1 week (n = 8) of ECMO support (**A**, RAS fingerprints). However, logarithms of PRA-S and aldosterone concentrations showed a better correlation after 1 week, whereby later deceased patients tended to exhibit a lower activity **(B)**. Aldo, aldosterone; Ang, angiotensin; PRA-S, plasma renin activity.

Continuous data on laboratory parameters are summarised in **Table 2**. Concentrations of endogenous cortisol and aldosterone were available for four patients pre-ECMO, for nine patients each within 24 h and after 1 week, and for six patients after 2 weeks. In three of all included patients, concentrations were available at all four observed time points. Concentrations were unavailable for one patient at one, for six patients at two, and for one patient at three time points. At all observed time points, median cortisol concentrations appeared to be lower than 275.86 nmol/L; wherefore, CIRCI was present in most of the observed individuals (**Figure 1A**). During ECMO support, cortisol was lowest during the first day of ECMO and slightly increased after 1 and 2 weeks. Concentrations of serum aldosterone showed a higher variability between individuals and were highest after 1 week of ongoing ECMO support (**Figure 1B**). **Figures 1C, D** show cortisol concentrations during vvECMO with respect to ARDS severity: at observed time points, most patients had moderate ARDS according to Horovitz index. Interestingly, overall values of both serum cortisol and aldosterone showed opposite trends, whereby cortisol tended to decrease, and aldosterone tended to increase with decreasing ARDS severity. Results of the RAAS subset analysis are depicted in **Figure 2**: Data of angiotensin metabolites were available for seven patients in the acute phase and for eight patients after 1 week of ECMO therapy, whereby angiotensin levels did not markedly differ between both time points (**Figure 2A**). Natural logarithms of PRA-S as marker of renin activity and aldosterone showed a better correlation after 1 week of ECMO support compared to the acute phase of ECMO (**Figure 2B**).

**Table 2:** Blood analyses and respiratory parameters at every observed time point.

	Pre-ECMO	Acute	One week	Two weeks	p.overall	N
	*N=4*	*N=9*	*N=9*	*N=6*		
Haemoglobin, g/dL	8.60 (8.55– 9.10)	8.80 (7.70– 9.10)	8.90 (8.80– 9.80)	8.30 (7.98– 8.78)	0.306	27
Platelet count, G/L	253 (206– 394)	213 (154– 224)	159 (100– 239)	164 (126– 203)	0.453	27
Leukocytes, G/L	9.07 (8.53– 11.7)	9.32 (8.81– 10.7)	8.58 (5.84– 11.2)	12.0 (9.11 –15.0)	0.776	27
**PTT, s**	**43.8 (41.6**– **45.8)**	**43.7 (37.3**– **47.3)**	**49.2 (45.1**– **60.6)**	**55.5 (53.1**– **70.3)**	**0.013**	**27**
Fibrinogen, mg/dL	1063 (798– 1105)	528 (509– 789)	600 (479– 645)	506 (425– 570)	0.262	27
CRP, mg/dL	23.9 (18.5– 24.2)	20.2 (17.2– 22.9)	9.74 (8.49– 21.2)	19.7 (19.2– 21.8)	0.406	27
IL-6, pg/mL	364 (259– 469)	151 (130– 226)	77.3 (23.6– 143)	131 (97.6– 195)	0.177	23
Procalcitonin, ng/mL	0.32 (0.26– 0.38)	0.85 (0.44– 1.01)	0.38 (0.29– 0.52)	0.51 (0.30– 1.08)	0.283	23
Serum creatinine, mg/dL	0.79 (0.69– 0.95)	0.72 (0.67– 0.94)	0.76 (0.65– 0.97)	0.92 (0.68– 1.46)	0.945	27
Blood urea nitrogen, mg/dL	30.3 (24.0– 36.9)	31.7 (28.8– 37.1)	35.5 (31.8– 40.0)	30.4 (25.7– 49.9)	0.856	27
Sodium, mmol/L	146 (146– 148)	147 (145– 150)	143 (139– 147)	144 (142– 146)	0.135	28
Potassium, mmol/L	4.70 (4.52– 4.80)	3.90 (3.70– 4.40)	4.20 (4.00– 4.60)	4.70 (4.38– 5.02)	0.052	28
Chlorid, mmol/L	106 (105– 108)	108 (106– 109)	106 (103– 111)	108 (103– 110)	0.764	28
Calcium, mmol/L	2.10 (2.09– 2.17)	2.05 (1.97– 2.14)	2.15 (2.12– 2.19)	2.14 (2.08– 2.18)	0.430	27
Magnesium, mmol/L	0.96 (0.80– 0.96)	0.91 (0.86– 0.98)	0.90 (0.89– 0.93)	0.86 (0.82– 1.01)	0.990	27
**Albumin, g/L**	**23.4 (22.5**– **24.1)**	**24.7 (22.7**– **25.4)**	**26.9 (26.1**– **30.9)**	**27.0 (26.4**– **30.2)**	**0.044**	**27**
ASAT, U/L	44.0 (38.0– 50.0)	48.0 (35.0– 54.0)	60.0 (55.0– 86.0)	46.5 (33.5– 56.5)	0.241	27
**ALAT, U/L**	**28.0 (25.0**– **39.0)**	**33.0 (27.0**– **43.0)**	**90.0 (67.0**– **95.0)**	**31.5 (28.0**– **42.5)**	**0.013**	**27**
γ-GT, U/L	467 (438– 484)	291 (124– 410)	898 (454– 1162)	406 (286– 638)	0.058	27
**LDH, U/L**	**278 (262**– **309)**	**390 (296**– **398)**	**443 (345**– **576)**	**470 (430**– **604)**	**0.013**	**27**
Total bilirubin, mg/dL	0.63 (0.61– 0.97)	1.15 (0.47– 1.32)	1.06 (0.64– 2.33)	1.26 (1.03– 1.40)	0.646	27
pO_2_, mmHg	66.0 (52.1– 88.8)	84.9 (78.2– 96.5)	78.9 (76.2– 85.2)	69.8 (66.3– 75.9)	0.186	28
**pCO_2_, mmHg**	**77.2 (71.6**– **85.0)**	**46.8 (42.8**– **51.9)**	**48.3 (41.7**– **50.3)**	**50.0 (48.0**– **52.5)**	**0.014**	**28**
BE, mEq/L	5.40 (4.20– 7.65)	7.30 (4.20– 9.50)	4.70 (3.50– 6.10)	3.70 (0.90– 7.47)	0.279	28
Lactate, mmol/L	0.60 (0.58– 1.02)	1.00 (0.80– 1.30)	1.20 (0.80– 1.40)	1.00 (0.62– 1.53)	0.725	28
Horovitz index (p_a_O_2_/F_i_O_2_)	87.8 (81.3– 124.0)	130 (104– 193)	127 (112– 137)	97.5 (79.9– 100)	0.179	28
**PEEP, cmH_2_O**	**13.4 (12.0**– **14.6)**	**13.1 (10.0**– **14.0)**	**11.3 (10.0**– **12.1)**	**8.00 (7.81**– **11.1)**	**0.040**	**28**
Ppeak, cmH_2_O	29.3 (28.7– 30.3)	26.1 (25.5– 27.1)	27.3 (25.9– 30.2)	29.9 (26.2– 31.8)	0.152	28
Respiratory rate	19.1 (15.8– 22.8)	12.0 (6.46– 13.8)	14.7 (13.3– 16.8)	20.0 (15.5– 24.1)	0.094	28
Tidal volume, mL	357 (330– 389)	231 (174– 302)	294 (194– 325)	208 (172– 292)	0.240	28
Cortisol, nmol/L	172 (93.3– 251)	114 (91.0– 194)	207 (96.9– 271)	215 (192– 322)	0.442	28
Aldosterone, pmol/L	6.65 (2.60– 83.1)	54.7 (21.6– 187)	93.3 (41.1– 129)	40.0 (10.0– 81.2)	0.255	28

All parameters are given as median (IQR). Bold parameters differed significantly between groups according to Kruskal–Wallis test (p.overall). Horovitz index, PEEP, Ppeak, respiratory rate, and tidal volume for each observation were recorded as the mean value observed over the respective day in each patient. ALAT, alanine aminotransferase; ASAT, aspartate aminotransferase; BE, base excess; CRP, C-reactive protein; IL-6, γ-GT, Gamma-glutamyltransferase; IL-6, Interleukine-6; LDH, Lactate dehydrogenase; pCO_2_, partial pressure of carbon dioxide; PEEP, positive end-expiratory pressure, pO_2_, partial pressure of oxygen; pPeak, peak inspiratory pressure; PTT, partial thromboplastin time.

### 3.3 Relationship between hypocortisolism, clinical parameters, and outcome

**Figure 3:**
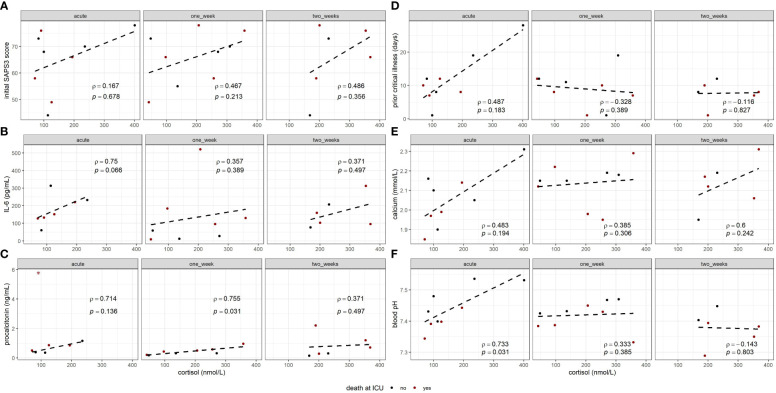
Correlation analyses between cortisol concentrations during vvECMO and clinical variables regarding patient’s condition, including initial SAPS3 score **(A)**, prior critical illness in days **(D)**, and blood laboratory parameters as IL-6 **(B)**, procalcitonin **(C)**, calcium **(E)**, and blood pH **(F)**.. Plots contain Spearman’s rho (ρ) and p-value (p) for each correlation. Initial SAPS3 score was assessed immediately before ECMO initiation. Prior critical illness refers to the duration of mechanical ventilation before ECMO. For procalcitonin (C, “acute”), a single outlier (*) was excluded from linear regression.

For separate time points during ECMO, linear regressions and Spearman coefficients between cortisol values and variables of interest are depicted in **Figure 3**. Although not significant, correlation with the initial SAPS3 score showed positive trends and fair Spearman coefficients after 1 and 2 weeks. Contrarily, correlations of cortisol with length of ICU stay (rho = −0.46, p = 0.213) and duration of vvECMO support (rho = −0.667, p = 0.059) indicated negative trends after 1 week of ECMO. Of all inflammatory and infection-related parameters, both IL-6 (rho = 0.75, p = 0.066) and procalcitonin (rho = 0.714, p = 0.136, single outlier excluded) correlated with cortisol during the acute state of ECMO support, a trend t hat, in case of procalcitonin, persisted after 1 week (rho = 0.755, p = 0.031). Investigating blood chemistry, cortisol concentrations showed marked positive correlations with blood pH (rho = 0.733, p = 0.031) and base excess (rho = 0.833, p = 0.008) in the acute state of ECMO, but not at later time points. To a lesser extent, similar observations could be made with calcium (rho = 0.483, p = 0.194) and the duration of critical illness before ECMO (rho = 0.487, p = 0.183), which did not show any associations to assessed adrenal function after 1 and 2 weeks. Regarding ECMO-specific parameters, ECMO gas flow showed a fair negative correlation with cortisol during the acute state of ECMO (rho = −0.583, p = 0.108). Other parameters that were significantly correlated with endogenous cortisol were albumin (rho = 0.717, p = 0.037) within the acute state of ECMO, lactate (rho = 0.692, p = 0.039), and potassium (rho = −0.787, p = 0.012) after 1 week and aldosterone (rho = −0.88, p = 0.021) after 2 weeks. Regarding outcome, no differences in either cortisol or aldosterone concentrations were observed between deceased and survivors at any time point.

## 4 Discussion

The present analysis revealed associations between adrenal function during vvECMO support and clinically observed parameters. Patients frequently presented with CIRCI throughout the observed period of 2 weeks, and total cortisol was associated with data regarding patient’s condition and inflammation. Since the pathophysiological mechanisms causing CIRCI during prolonged ARDS are yet to be determined, our findings represent novel insights into the connections of hypoadrenalism to sufficiency of oxygen supply and disease severity. Given the complexity of an accurate interpretation in the state of critical illness, the study design enabled to assess adrenal function in a homogeneous collective of glucocorticoid- naive patients with COVID-19 ARDS, a disease that eminently served as model for the investigation of respiratory failure.

### 4.1 Prolonged respiratory failure as a model for CIRCI development

As seen in patients with trauma or septic shock, the presumed cause of CIRCI is often related to the underlying disease (12). In the context of prolonged ARDS, however, the connection between hypoxia and CIRCI remains to be determined. The adrenal glands of calf respond to acute hypoxia, considered as stressor, by the prompt secretion of cortisol into the circulation (28). On the other hand, the response to ACTH has been shown to be decreased in mice under hypoxic conditions (29), illustrating an uncoupling of the HPA axis, as it was stated in prolonged critical illness (30). Furthermore, in humans with chronic obstructive pulmonary disease, hypoxic conditions were also related to low cortisol values (31).

In our collective, vvECMO can be seen as the effort to improve oxygenation during the concomitant evaluation of adrenal function. All our patients presented with low cortisol concentrations throughout the observation period, whereby values were lowest after ECMO initiation. Especially in the acute state of ECMO, low cortisol concentrations might therefore indicate still persistent adrenal compromise due to the preceding duration of critical illness. However, cortisol concentrations progressively increased during ECMO, wherefore oxygenation support might result in a partial rehabilitation of adrenal function over time. Of note, a reduction in cortisol breakdown might also explain the observed increase in cortisol levels, which has been previously described by Boonen et al. in patients with critical illness (32). Nevertheless, most of our patients already experienced prolonged critical illness for at least 1 week previous to study inclusion, which is different to the population investigated by Boonen et al.

Similarly, aldosterone values appeared to be highest after 1 week, and the conducted sub-analysis indicated a more homogeneous aldosterone response to PRA-S after 1 week of ECMO compared to the acute phase. The relevance of hyperreninemic hypoaldosteronism in respect of hypoadrenalism during critical illness remains controversial (4). Nevertheless, the hereby observed trends may be of interest for future research within critical illness.

### 4.2 The adrenal response to extracorporeal life support

Presently, there is only limited information about the direct impact of ECMO on HPA axis signalling. In a setting of low-dose GC treatment and alternative veno-venous- to veno-arterial ECMO support, Altshuler et al. reported higher cortisol and ACTH levels in deceased patients compared to survivors (7), and pre-ECMO conditions were investigated from Agus et Jaksic, which showed low cortisol and aldosterone values in a small collective of ECLS primed circuits of infants (33). As a drastic measure of organ replacement, ECMO acts as a stressor via haemodynamic changes, inflammatory and coagulopathic effects due to cannulation, predisposition to infection, or bleeding complications (7, 18). Therefore, ECMO support itself might be a reason for additional alterations in HPA-axis signalling.

In the acute phase, cortisol also correlated with IL-6 and procalcitonin. These correlations may express the adrenal stress response to ECMO initiation itself, which may instigate the adrenal reaction to already present stimuli, such as inflammation and concomitant bacterial infection, by conveying an additive effect (19). Further contemplating the acute phase of ECMO, cortisol also showed positive correlations with the previous duration of critical illness, total calcium, pH, and base excess. Even if ionised calcium did not show any correlation with cortisol, and CIRCI is normally related to mild hypercalcaemia (3), dependencies of calcium homeostasis on the cortisol-mediated stress response seem reasonable (20). Alkalosis is a reported complication of ECMO (21). Thus, base excess levels are also likely to be initially elevated in a manner of post-hypercapnic alkalosis (22). However, to our knowledge, this is the first report that relates post-hypercapnic alkalosis to the HPA stress response after ECMO initiation. Future prospective investigations may provide more information to optimise the management of acid–base balance within the acute state of ECMO.

### 4.3 Severe ARDS: lessons learned from COVID-19

Compared to classical non-COVID ARDS, COVID-19 ARDS is characterised by distinct features of the inflammation response and the prominent RAAS dysregulation (8, 23). A direct impact of COVID-19 on the adrenal cortex was proposed by Mao et al., who also reported lower cortisol levels of critically ill patients with COVID-19 compared to critical illness of other causes (6). However, unlike their control subjects, the majority of COVID-19 patients received veno-arterial ECMO, which might also be associated with low cortisol concentrations.

Especially in COVID-19-ARDS, the duration and severity of critical illness before ECMO are intensely discussed outcome parameters. A recent meta-analysis reported a longer duration of IMV and longer duration of symptoms before vvECMO initiation to be associated with increased mortality (24). However, adjusted analysis of IMV duration before ECMO showed a wide variation in effect size, relativising this finding, as it was previously reported by our study group (9). The observed correlation between cortisol and prior IMV duration may provide further insight into the adrenal response to ECMO according to the previous duration of critical illness.

Alterations of the alternative RAS were previously reported in severe COVID-19 ARDS (23).

Throughout the observation period, cortisol values of our participants tended to be positively associated with initial SAPS3 and negatively associated with both length of ICU stay and duration of ECMO support. Furthermore, cortisol was highest in severe ARDS according to Horovitz index, indicating a rest function of the adrenal response to disease severity. Those findings differ to non-ICU patients (25). No difference in cortisol between survivors and non-survivors was observed. However, especially in COVID-19, the role of serum cortisol as predictor for survival is still unclear (26); wherefore, further studies will be needed to clarify the relation between adrenal function during prolonged severe COVID-19 ARDS, disease severity, and survival.

Within our population, neither a discrepancy in corticosteroid insufficiency between COVID-19 patients with and without metabolic disorders (i.e., diabetes, obesity) nor correlations between cortisol and BMI could be observed. As a manifestation of HPA-axis disturbance, the state of CIRCI may bias the given relations between endogenous cortisol and markers of body composition (27). Nevertheless, because of the known associations between metabolic disorders and COVID-19 severity (34, 35), future comparisons of long-term HPA axis signalling in critical illness to BMI and estimators of glycaemic control may reveal new insights into the metabolic adaptations during CIRCI.

## 5 Limitations

Considering the retrospective manner and the high adherence to current treatment guidelines including corticosteroids, the study was restricted to a small sample size. It is important to note that this descriptive data analysis aimed to provide initial insights in the relevant topic of oxygenation- related HPA recovery, and the limited sample size emphasises the need for cautious interpretation of our findings. However, according to our restrictive in clusion and exclusion criteria, we were able to observe unbiased total cortisol values of the same patients at multiple occasions along a predefined timeline; wherefore, we considered our data eligible to determine adrenal insufficiency during this critical condition. The lack of a control group consisting of critically ill COVID-19 patients without ECMO support further restricts our study to its descriptive character. The design of a control group in a prospective setting might be challenging, since the time point of study inclusion would be difficult to compare between groups. Nevertheless, the implementation of a control group to investigate ECMO related HPA axis signalling with a comparable inclusion time point may contribute to an increase in explanatory power of future studies. Regarding the duration of critical illness, there was no present information of initial ICU admission, since most patients were transferred from other ICUs to receive ECMO support at our centre. Thus, we considered previously reported invasive mechanical ventilation as time frame of critical illness before ECMO, according to which most patients were in the chronic phase of critical illness.

Due to the low number of observations in which patients presented without CIRCI during ECMO, significant differences between parameters of CIRCI and non-CIRCI were not considered reliable. Moreover, a direct comparison between groups would be beyond the scope of this descriptive analysis but should be separately evaluated in future studies.

## 6 Conclusions

The impairment of adrenal function in the course of prolonged critical illness has been frequently related to conditions complicating patient management. Our data indicate benefits of oxygenation support for adrenal function in prolonged COVID-19 ARDS and provide additional information on the necessity of glucocorticoids during vvECMO support. If a partial recovery of the adrenal glands after the improvement of oxygenation can be expected in similar populations, and if cortisol might subsequently serve as marker for the adrenal response to disease severity in those patients demand further evaluation. However, the investigation of hypoxia treatment via vvECMO as indicator for adrenal rehabilitation in CIRCI marks a novel approach, which might be of use in future studies to further determine the influence of oxygenation support on adrenal function. Considering ARDS, finding a causality between each respective underlying disease and CIRCI development may prove as another important step to individual patient management.

## Data availability statement

The raw data supporting the conclusions of this article will be made available by the authors, without undue reservation.

## Ethics statement

The study was approved by the ethics committee of the Medical University of Vienna (#1694/2018). The studies were conducted in accordance with the local legislation and institutional requirements. The human samples used in this study were acquired from primarily isolated as part of your previous study for which ethical approval was obtained.Written informed consent was obtained.

## Author contributions

CB: Conceptualization, Data curation, Investigation, Methodology, Software, Visualization, Writing – original draft, Writing – review & editing. PW: Conceptualization, Funding acquisition, Investigation, Project administration, Supervision, Writing – review & editing. AH: Data curation, Writing – review & editing. SK: Data curation, Writing – review & editing. MM: Data curation, Writing – review & editing. DL: Data curation, Writing – review & editing. MP: Visualization, Writing – review & editing. OD: Visualization, Writing – review & editing. KK: Formal Analysis, Supervision, Visualization, Writing – review & editing. JS: Formal Analysis, Writing – review & editing. AK-W: Writing – review & editing. MK: Formal Analysis, Supervision, Writing – review & editing. MH: Conceptualization, Data curation, Formal Analysis, Funding acquisition, Methodology, Resources, Supervision, Validation, Writing – review & editing.
